# Overlay Tool^©^ for aCGHViewer^©^: An Analysis Module Built for aCGHViewer^©^ used to Perform Comparisons of Data Derived from Different Microarray Platforms

**Published:** 2007-08-08

**Authors:** Ken C. Lo, Ganesh Shankar, Yaron Turpaz, Dione Bailey, Michael R. Rossi, Tania Burkhardt, Ping Liang, John K. Cowell

**Affiliations:** 1 Department of Cancer Genetics, Roswell Park Cancer Institute, Buffalo, NY 14263; 2 Affymetrix, Inc. Santa Clara, CA 95051; 3 Yale University School of Medicine, Department of Cancer Genetics, New Haven, CT 06520

**Keywords:** Overlay Analysis, Microarray, ACGH, expression profiling, CNAs, aCGHViewer

## Abstract

The Overlay Tool^©^ has been developed to combine high throughput data derived from various microarray platforms. This tool analyzes high-resolution correlations between gene expression changes and either copy number abnormalities (CNAs) or loss of heterozygosity events detected using array comparative genomic hybridization (aCGH). Using an overlay analysis which is designed to be performed using data from multiple microarray platforms on a single biological sample, the Overlay Tool^©^ identifies potentially important genes whose expression profiles are changed as a result of losses, gains and amplifications in the cancer genome. In addition, the Overlay Tool^©^ will incorporate loss of heterozygosity (LOH) probability data into this overlay procedure. To facilitate this analysis, we developed an application which computationally combines two or more high throughput datasets (e.g. aCGH/expression) into a single categorized dataset for visualization and interrogation using a gene-centric approach. As such, data from virtually any microarray platform can be incorporated without the need to remap entire datasets individually. The resultant categorized (overlay) data set can be conveniently viewed using our in-house visualization tool, aCGHViewer^©^ (Shankar et al. 2006), which serves as a conduit to public databases such as UCSC and NCBI, to rapidly investigate genes of interest.

## Introduction

Cytogenetic abnormalities are consistently associated with the development of the tumor phenotype and have been used extensively as diagnostic and prognostic tools (Sandberg, 1980). The development of Comparative Genome Hybridization (CGH) by Kallionemi et al. (1992) provided a cost-effective and efficient method of identifying copy number aberrations (CNAs) across the entire genome without the need for metaphase chromosomes from the tumor cells. With the recent evolution of CGH onto a microarray platform (aCGH), large-scale high-resolution studies can now be performed with small DNA samples, accelerating the discovery process for molecular mechanisms of tumorigenesis and progression. One aCGH platform that is now widely used involves large insert clones such as bacterial artificial chromosomes (BACs), although oligonucleotide arrays have also been developed (Carvalho et al. 2004).

One of the confounding problems of using these aCGH approaches to study CNAs on a genome wide basis is that it is often difficult to discern those losses/gains/amplifications that are ‘real’ discoveries from artifacts introduced either by the technology or as a result of, for example, mismapped BACs (Rossi et al. 2005) or BACs that are located in regions that are inherently polymorphic for copy number (Iafrate et al. 2004). In an attempt to resolve these issues, numerous statistical approaches have been developed to assess the relative copy number in each segment of the genome, while controlling the false discovery rate (FDR). These approaches include Hidden Markov Model, Circular Binary Segmentation (CBS), quantile smoothing, Bayesian, adaptive weights smoothing, clustering, and heuristic smoothing methods (Olshen et al. 2004; Eilers and Menezes, 2005; Daruwala et al. 2004; Hupe et al. 2004; Wang et al. 2005; Jong et al. 2004). While these methods accurately identify abnormal regions, the aCGH platform can only provide insights into copy number aberrations. With the discovery of these aberrations, numerous follow-up questions are raised. For example, when a segment of the genome is amplified/deleted, what are the biological consequences? Generally, when a segment containing a known oncogene is amplified, the resulting amplification presumably leads to an overexpression of that oncogene, driving tumor growth/progression. In most circumstances, these observations are confirmed independently using non-array based gene expression verification. However, since large segment CNAs contain many genes, prioritizing which ones to verify can be difficult. Ideally, given unlimited resources and manpower, all genes within amplifications/deletions would be independently verified, but this is a time consuming and labor-intensive endeavor that is often cost-prohibitive on a gene-by-gene basis.

Another platform that is widely adopted to study genes associated with cancer involves gene expression profiling using oligonucleotide arrays. Essentially, genes associated with tumorigenesis can be identified by comparing expression patterns between normal and tumor samples. These arrays allow the simultaneous evaluation of gene expression levels of over 54,000 probe sets (using the Affymetrix GeneChips ^®^ U133 Plus 2 array, for example) in a single experiment. As with aCGH, statistical methods such as analysis of variance (ANOVA) (reviewed by Cui and Churchill, 2003), as well as Significant Analysis of Microarrays (SAM) (Tusher et al. 2001), and Principle Component Analysis (PCA) (Yeung et al. 2001) have been used to eliminate genes which either do not show significant changes or are not associated with the hypothesis. Again, the question remains whether the gene expression variation reflects true biological differences, or systematic noise? The problem is more significant for the Affymetrix platforms since generation of this data does not use comparative co-hybridization with matching normal samples to control for variation. Thus, small fluctuations during the various stages of establishing the gene expression profile may introduce additional noise into the system. To overcome this problem, Affymetrix has incorporated several design features such as the Perfect Match/Mismatch probe sets to lower the FDR, as well as the development of new statistical analysis algorithms to increase the robustness of normalization and analysis. Despite these advancements, a typical experiment will generally identify statistically significant candidate genes that are too numerous to be verified individually.

One way to assess the relative importance of gene expression changes is to combine the analysis of two different platforms from the same biological samples. This overlay analysis can potentially identify genes within specific chromosomal regions that demonstrate CNA or loss of heterozygosity (LOH) with corresponding increases or decreases in expression, thereby providing a filter to determine the ‘drivers’ of the CNA/LOH. This follows since it is perceived that tumors gain increased malignancy as a result of acquiring selective chromosomal gains/losses, or LOH, as mechanisms for altering gene function/expression. The underlying advantage of this approach is the high throughput identification of potential changes in gene expression with associated mechanism, which then increases the robustness of either platform by itself.

Due to the data-intensive nature of each of the high-throughput platforms, computational overlay of datasets required the development of specific software to achieve our goal. To this end, we have developed an application that can combine multiple datasets by establishing agreement between complementary platforms. This program outputs a tab-delimited text file to be used with our in-house visualization tool, aCGHViewer^©^ (Shankar et al. 2006), as well as providing access to the raw data through a summary user-interface.

## Results

### Development of the Overlay Tool^©^

At the time of preparation of this manuscript, no standard approach for combining data from aCGH and expression arrays was publicly available. The biological question we are attempting to address focuses on selective pressure gained within the confines of a copy number aberration or region of LOH. For example, when an amplification event is observed, which genes in the amplicon are upregulated that can be investigated for their potential to confer growth advantage to a tumor cell? Recently, work in our group has demonstrated that, within a given amplicon, only certain subsets of the genes within that region are upregulated (Rossi et al. 2005). The assumption is that only ‘drivers’ that contribute to tumor growth/progression are overexpressed (and selected for) in an amplicon, while bystanders are not overexpressed since they have no selective advantage (or were simply not actively expressed in that tissue in the first place). Due to the data-intensive nature of high throughput technology, however, manually combining the raw data between the two platforms became too labor intensive to perform even on relatively small sample sizes. To overcome this limitation of data analysis, we developed an analysis protocol implemented in Microsoft^®^ Visual Basic 2005^®^ to computationally combine the two datasets and assign a category type based on agreement/disagreement between the multiple datasets from the aCGH, oligonucleotide based expression platforms and oligonucleotide based SNP arrays. This tool has been named the RPCI Overlay Tool^©^™.

As part of the developmental process for the aCGHViewer^©^ (Shankar et al. 2006), the Overlay Tool^©^ is the first analysis application designed for the aCGHViewer^©^ pipeline, which takes advantage of the category flagging capability inherent in aCGHViewer^©^ to highlight major changes when agreement is found between datasets. In this case, overexpressed genes caused by amplification, or down-regulated genes caused by deletions, are denoted as significant changes and are displayed in aCGHViewer^©^ as red (gains/overexpression) or green (losses/downregulation).

### Implementation and features

The Overlay Tool^©^ is implemented as a stand-alone application, with a tab-delimited output that is specifically designed to be visualized within the aCGHViewer^©^ (Shankar et al. 2006). Currently, the Overlay Tool^©^ requires at least 128Mb of RAM, Microsoft^®^ .NET Framework 2.0, Microsoft^®^ Windows 2000^®^ operating system or above, and J2SE^®^ 1.3 or greater (full install, not just the JRE for aCGHViewer^©^) to run. The option to query the raw data (at the summary level) is achieved via a summary user interface, with an option to invoke an embedded version of the aCGHViewer^©^. The design is such that the viewer may be used for external database queries, while interrogating the Overlay Tool^©^ categorization and the underlying raw data. After confirming the call results, based on the user-selected stringency, the analysis can be saved and the data re-analyzed using different options. An internet connection is required to launch queries against public databases such as UCSC Genome Browser via the hyperlinks displayed in the summary window.

**Figure 1.**
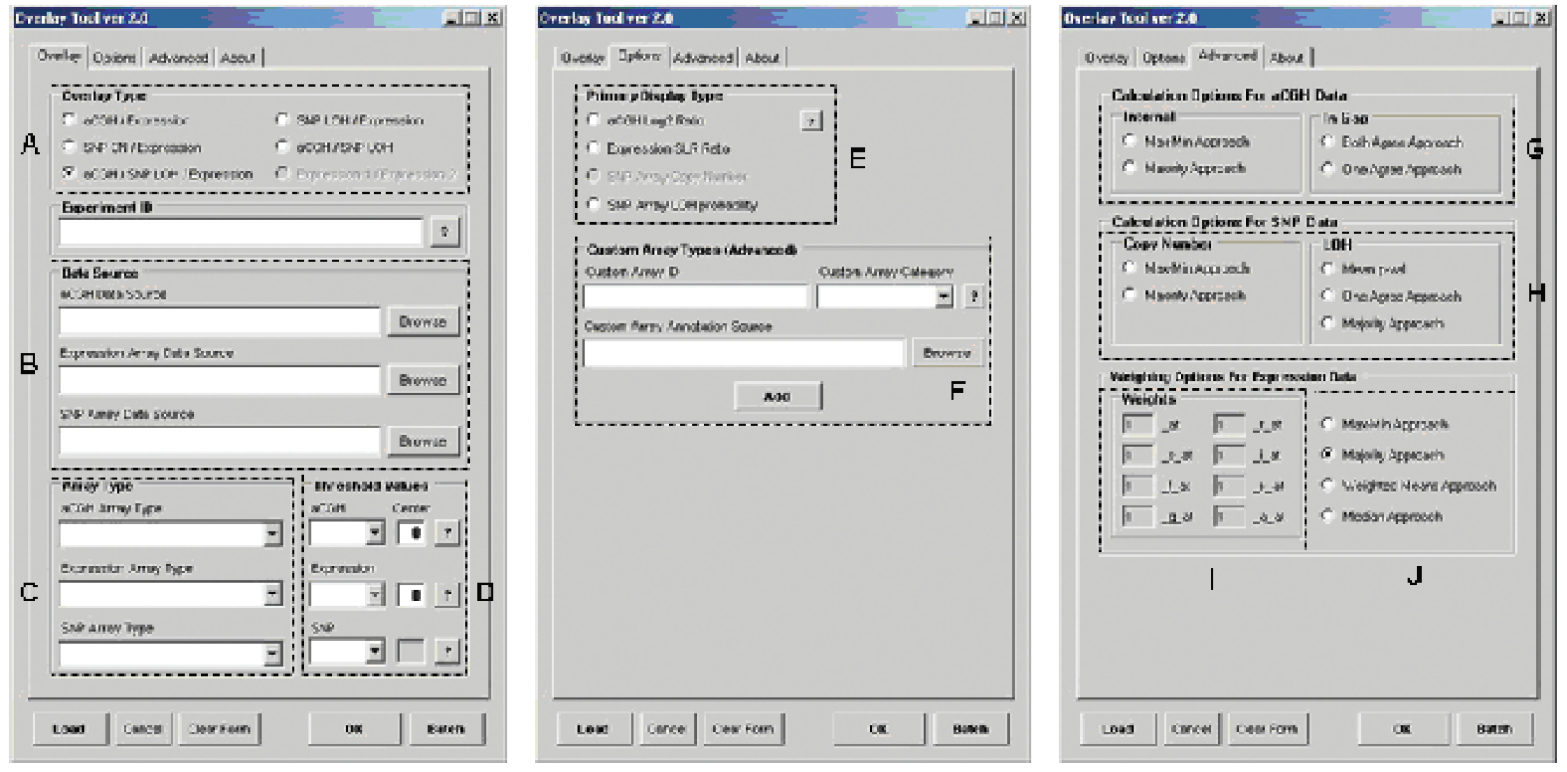
The graphical user interface for the Overlay Tool^©^. This interface allows the choice of overlay type and the array platforms to be selected, as well as user-defined threshold values. The various sections as outlined (dotted lines) are designed for user input of the following information: (**A**) Overlay Type, (**B**) Data Source for array data, (**C**) Array Platform type, (**D**) User-defined threshold and Data Center, (**E**) Primary Display Type, (**F**) Option for users to add Custom Array Types to the Overlay Tool^©^, (**G**) Calculation options for aCGH data, (**H**) Calculation options for SNP array data, (**I**) Weighting scheme based on probe set suffixes for the Affymetrix gene expression arrays and (**J**) Calculation options for Affymetrix gene expression arrays.

**Figure 2.**
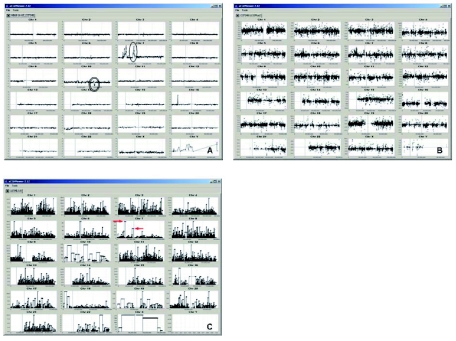
The aCGHViewer^©^ genomic view of the RPCI 6K BAC Array (**A**) showing the graphical representation of aCGH data from a malignant glioblastoma. Notice amplification of the *EGFR* locus (circle) on chromosome 7, a homozygous deletion around *PTEN* locus on chromosome 10 (double circle) and a single copy loss of chromosome 10, which are common cytogenetic events associated with glioblastoma. In (**B**), the aCGHViewer^©^ genomic view of the SLR values from Affymetrix U133Plus2 expression array of the same glioblastoma sample is shown. Due to the intra data noise levels, it is difficult to establish the relationship between the two datasets (**A** and **B**) based on visual inspection alone. In **C**, the aCGHViewer^©^ genomic view of the LOH p-values of the Affymetrix Mapping 100K SNP Array of the same glioblastoma sample is shown. Examples of regions showing high confidence of LOH based on pooled normal allelic frequencies are highlighted by arrows (not all areas are highlighted to avoid clutter). Note the Y-axis scale differs for each chromosome depending on the range of –log p-values which is important for interpretation of LOH (see text).

**Figure 3.**
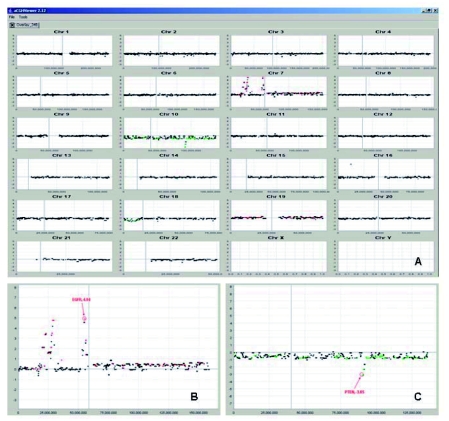
The graphical display of the overlay between aCGH (from the RPCI 6K BAC Array) and gene expression data (from Affymetrix U133Plus2 Array) viewed by aCGHViewer^©^ (**A**). Notice that numerous data points have either been colored red or green, signifying concordance between the two platforms. Red spots signify genes that show both a gain in copy number using the BAC array and upregulation using the U133Plus2 array. Green spots signify genes that show a loss in copy number by the BAC array coinciding with downregulation by the U133Plus2 arrays. For more details of the various types of overlay and the different interpretation of the highlighting scheme, see table 3. In (**B**), the Overlay Results displayed by the aCGHViewer^©^ for chromosome 7 are shown. Red/Green spots represent concordance between the aCGH and expression platforms. *EGFR* has been annotated using the aCGHViewer^©^ annotation tool. In (**C**), the overlay results for chromosome 10 are shown. *PTEN* is annotated using the same annotation tool. In both (**A**) and (**B**), the primary display data are log 2 ratios from the BAC array analysis. Notice that both *EGFR* and *PTEN* were highlighted by the Overlay Tool^©^.

The Overlay Tool^©^ currently allows analysis of datasets from three categories of microarrays: 1) copy number changes from either BAC arrays or Copy number prediction from the Affymetrix GeneChip ^®^ Mapping SNP arrays, 2) Gene expression profiling from the Affymetrix GeneChip expression arrays and 3) LOH probability from the Affymetrix GeneChip ^®^ Mapping SNP arrays. However, the Overlay Tool^©^ is constructed to support data from virtually any custom array type provided that it is supplied with a platform-specific annotation file compatible with the Overlay Tool^©^ (Fig. 1F). Currently, the Overlay Tool^©^ supports five types of overlay: 1) aCGH/expression, 2) SNP copy number prediction/expression, 3) aCGH/ LOH probability, 4) LOH probability/expression and 5) aCGH/LOH probability/expression (Fig. 1A). To avoid clutter, and data overload to the user, the output of the Overlay Tool^©^ displays only one primary data type dictated at the user’s discretion (Fig. 1E), with concordant changes in the complementary platform highlighted by changing the data points either red or green allowing easy visualization (Figs. 2 and 3).

**Figure 4.**
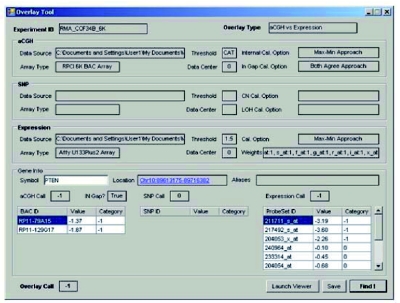
The summary graphical interface that provides access to the various data points used to generate the cross platform category information, as well as providing the option to launch aCGHViewer^©^. The Interface also displays the user-defined settings used to generate the overlay. A hyperlink is added to the UCSC genome browser for more detailed information.

The Overlay Tool^©^ also provides multiple, user-adjustable, settings which allows individual experience/expertise to dictate the stringency used when determining concordance and discordance between datasets (Fig. 1G, 1H, 1I and 1J). Once the comparisons are completed, a graphical user interface allows the data that gave rise to the concordance/discordance call to be queried by entering the gene symbol, as well as launching the aCGHViewer^©^ for graphical display of the genome (Fig. 4).

### Processes of the Overlay Tool^©^

**Figure 5.**
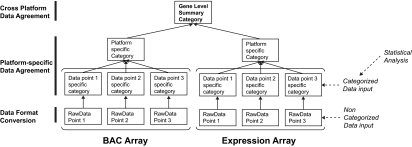
Schematic summary of the categories generated by the Overlay Tool^©^ for each gene. Category data is generated at three levels (data point-specific category, platform-specific category and gene level summary category). Where statistical analysis is performed on a particular platform, the statistical category data selected can be used in the data point-specific category instead of comparing to a user-defined threshold by choosing the “CAT” option in user-defined threshold.

At the core, the Overlay Tool^©^ consists of three major parts: Data Format Conversion, Platform-specific Data Agreement and ‘cross platform data agreement’ (Fig. 5).

### Data format conversion

Since each platform has its own unique annotation methods and identifiers, it was necessary to implement a data format conversion step to facilitate the comparison procedures. For example, the epidermal growth factor receptor (*EGFR*) gene is represented by two different BACs on the RPCI 6K BAC array, 15 Probe Sets on the Affymetrix U133 Plus 2 array and 29 SNPs on the Affymetrix Mapping 100K SNP array platforms (Table 1). To overcome this diversity, we have converted all annotation for a single gene into one descriptor—the gene symbol. The input data is then converted using the built-in Overlay Tool^©^ annotation files. Thus, *EGFR* now becomes the new identifier which allows data from virtually any source to be incorporated. For example, all of the associated data points for *EGFR* would then be distilled into two data sets evaluated under the ‘*EGFR*’ identifier (Table 1 and 2).

Annotation files for the BAC arrays are generated by using the FISH mapping and BAC end sequence information (available at http:/hgdownload.cse.ucsc.edu/goldenPath/hg18/database/) and comparing them to the NCBI published positional information of the known genes determined by Refseq alignment, protein evidence and/or transcript evidence. In our specific application, a minimum 30% of the gene sequence coverage is required to associate a BAC to a gene, which avoids erroneous assignment of a gene to a BAC with minimal overlap.

The associated gene symbol for the Affymetrix probe set IDs were used to generate the annotation files for the Affymetrix platforms after converting them to the HUGO-approved unique identifiers. This process facilitated identifying those particular Affymetrix probe sets that used either a previous HUGO symbol or alias as its main identifier. Whenever a particular probe set points to a gene symbol that is not unique (either the symbol is both a previous symbol of another gene and a current approved symbol in the HUGO database), the mapping information provided by Affymetrix is cross-compared to the HUGO database to manually resolve the accepted identifier. With the Affymetrix mapping SNP arrays, only SNPs that are located within 10,000bp of the genomic location of the start or the end of a gene are used. Although the mapping SNP arrays also provide an estimate of copy number at each locus, due to the noise levels associated with the SNP array, we felt it was not appropriate to use similar inference type evaluations that have been applied to the BAC arrays, although the high density nature of the SNP arrays provide a more extensive coverage than the BAC arrays (see below).

### Platform-specific data agreement

#### BAC arrays

During the development of the Overlay Tool^©^ strategy using the gene-centric approach described above, it was necessary to overcome the problem that many genes were located in regions of the genome that were not represented by a specific BAC on lower resolution arrays. As BAC arrays become more comprehensive, however, this is becoming less of a concern, although many existing datasets will still have been derived from lower density arrays. To address this issue, genes are placed in one of two classes (Internal and in Gap) depending on the coverage of the aCGH platform. The different category genes are evaluated by different mechanisms. From our experience with BAC arrays, we found that the noise level was acceptable to evaluate these ‘in Gap’ genes by essentially inferring the copy number at that locus based on the two neighboring BACs present on the array.

During the analysis, the Overlay Tool^©^ imports the necessary raw data from the datasets and evaluates each data point by comparing this value to the user-defined threshold (for non-categorized data) and assigns a data point-specific category of 1 (gain), −1 (loss) or 0 (no change). For categorized data, the categories generated by other statistical analysis tools are used instead of performing a comparison with the user-defined thresholds (Fig. 5).

#### ‘I’ Class

**Figure 6.**
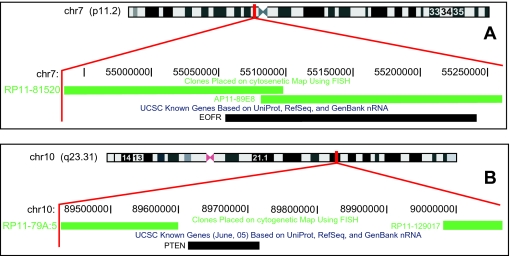
(**A**): Genomic location and cytogenetic information of the *EGFR* gene, a category ‘I’ gene in the RPCI 6K BAC array. Notice *EGFR* is covered by two BACs (RP11-81B20 and RP11-89E8). (**B**) Genomic location and cytogenetic information of tumor suppressor *PTEN*, a category ‘G’ gene in the RPCI 6K BAC array. Notice *PTEN* is flanked by two specific BACs on the RPCI 6K BAC array: RP11-79A15 and RP11-129G17.

The ‘I’ class consists of genes for which at least 30% of the coding region is represented by BACs on the particular array used (Fig. 6). When multiple BACs (aCGH) on the array represent the signal for a particular gene, the data point-specific categories described above are further evaluated according to the user-defined choice of calculation method to give a platform-specific summary category (see Fig. 5). The current version of the Overlay Tool^©^ has two options for the ‘I’ class: a ‘max-min’ approach and a ‘majority’ approach. Both the ‘max-min’ approach and the ‘majority’ approach are evaluation methods that generate the platform-specific summary category data based on data point-specific categories. If the ‘max-min’ approach’ is selected, only one of the data point-specific summary categories within the series needs to show deviation from ‘no change’ to define this gene as either gained or lost in the platform-specific summary category. This approach is more liberal than the ‘majority’ approach, where the number of data points that have categories of gain/loss must outnumber their opposite counterpart to change the platform-specific summary category to represent that trend.

#### ‘G’ Class

Unlike the ‘I’ class genes, ‘G’ class genes are not specifically interrogated by a specific BAC on the array. Due to the consistently high signal to noise ratio, an evaluation is based on ‘inferred’ data from the neighboring BACs. ‘G’ class genes lie in regions of the chromosome which are not covered by a specific BAC. However, if two adjacent BACs on the array show a CNA, it is inferred that the region between them follows the same trend (gain or loss). For example, the locus containing the tumor suppressor gene, phosphatase and tensin homolog (*PTEN*), is deleted in a small proportion of malignant glioblastomas (Fig. 6). The *PTEN* gene sequence is not specifically represented on the RPCI 6K BAC array but is flanked by BACs RP11-79A15 and RP11-129G17. The values of the two neighboring BACs are first evaluated either by comparison to user-defined thresholds (non-categorized data) or by using previously generated categories through statistical analysis (categorized data) to give each a data point-specific category. The platform-specific summary category is then evaluated by either one of two currently supported approaches: One-Agree approach or Both-Agree approach. When the ‘Both-Agree approach’ is selected, the platform-specific summary category deviates from ‘no change’ only when both of the neighboring BACs have concordant data point-specific categories of gain/loss. This is contrasted by the One-Agree approach where any one of the two BACs showing a trend will generate a platform-specific summary category of that trend. In our opinion, this evaluation method can be dangerously liberal, and while we recognize that this approach may not be appropriate in all circumstances, we have included this option to be used solely in highly specific scenarios. Since we have found that the Both-Agree approach may lead to the erroneous exclusion of genes residing in gaps adjacent to large amplifications, the use of the One-Agree approach is valid with the caveat that it is applied in the appropriate manner.

### Affymetrix gene expression arrays

As the Affymetrix GeneChip platform has evolved, the accompanying analysis tools have also changed. Various statistical methods have been used throughout the analysis process to assess differential expression between test and reference samples (i.e. tumor/ normal). While some of these tools are generally geared towards analysis at the individual probe level, a design decision was made during the development of the Overlay Tool^©^ to analyze data from the probe set summary level. This approach provides the flexibility to apply various background correction, outlier detection and normalization algorithms.

Once the appropriate signal log ratio (SLR) is generated between test and reference, the Overlay Tool^©^ first evaluates each probe set individually and generates a data point-specific category exactly as it does for the BAC array. However, during generation of the gene level summary category, there are more user-tunable parameters available to adjust the stringency of the analysis of expression data. These include: 1) ‘max-min’ approach, 2) ‘majority’ approach, 3) ‘weighted means’ approach and 4) ‘median’ approach, as well as the user-defined weighing scheme based on probe set suffixes (Fig. 1I and 1J).

Since evaluation approaches (1) and (2) have been explored earlier, only options (3) and (4) warrant clarification here. In the higher density Affymetrix arrays, individual genes are often represented by multiple probe sets. In any given analysis, it is not uncommon that the probe sets for a given gene will show variation in SLR values and sometimes show an opposite trend. It was important, therefore, to provide a mechanism to accommodate these eventualities. To reconcile this, aside from allowing the same evaluation methods for gene-level summary categorization that were available in the BAC array, we developed additional methods to use the ‘center’ (either by way of a mean or median) as the value to determine the gene-level summary category. These methods differ from the previous two in that both the ‘max-min’ and ‘majority’ approach evaluate gene expression levels by looking for outliers, whereas the ‘weighted means’ or ‘median’ approach require the ‘center’ to pass the user-defined threshold for a summary category representing that trend to be generated. These methods are considerably more stringent than either the ‘max-min’ or ‘majority’ approaches, and should be used only when this stringency is deemed to be appropriate.

In addition to the various summary category generation methods, a user-defined weighing scheme can be used based on user experience/ expertise. The Overlay Tool^©^ provides the opportunity to weight groups of probe sets (by suffixes) differently, so that if a particular group of probe sets have been found to be unreliable, they can effectively be removed from the platform-specific summary category consideration. For example, an Affymetrix probe set ending with ‘_x_at’ on the U133Plus2 platforms, signify that this group of probe sets may cross-hybridize in an unpredictable manner (Affymetrix Data Analysis Fundamentals, 2005). If it is desirable to discount these probe sets from further analysis, then a weigh of ‘0’ to the ‘_x_at’ probe sets can be entered into the user-defined weighing scheme (Fig. 1I). In this case, none of the probe sets ending with ‘_x_at’ will be considered during platform-specific summary category consideration.

### Affymetrix mapping SNP arrays

For overlay analysis, only data points that are located within 10 kb of the start and end of the genomic location of a gene are used for evaluation in the Overlay Tool^©^. Based on our experience with the noise level of the mapping SNP array platform, it was not considered appropriate to use similar evaluation mechanisms for the genes that fall into gaps between data points, as described earlier for the BAC arrays. Thus, currently, only genes that have direct coverage by the SNP array are evaluated and considered in this application.

The Affymetrix Mapping SNP Arrays can generate two different categories of data: copy number based on the Copy Number Analysis Tool (CNAT) and a LOH probability at each SNP location based on pooled normal allelic frequencies. The copy number data generated from the mapping SNP arrays are treated in ways similar to that described for the BAC arrays (with the exception discussed above), for which both ‘max-min’ and ‘majority’ approaches are available. The nature of the LOH probability data generated from the mapping SNP array, on the other hand, differs from the copy number data in that it displays the inverse probability that LOH has occurred at any particular locus. This data is treated in a similar way, in that each data point is compared to the user-defined thresholds to generate the data point-specific category. After all data points are evaluated, the platform-specific summary category is generated by one of three evaluation methods: 1) mean p-val, 2) ‘one agree’ approach and 3) ‘majority’ approach (Fig. 1H). A mean p-value approach is similar to the ‘weighted means’ for expression, in that a calculation of the mean of all the gene-specific data points is performed before a comparison with the user-defined threshold. The platform-specific summary category for LOH is evaluated either as 1 (likely shows LOH) or 0 (unlikely to show LOH). Unlike the ‘one agree’ and ‘majority’ approaches, the mean calculation uses the ‘center’ to gauge whether this is a good likelihood of LOH or not. However, one note of caution is that the default setting for the Affymetrix Copy Number Analysis Tool (CNAT) for genome smoothing is 0.5 Mb, which is larger than the average length of a gene. Thus, smoothing using this default setting may lead to over dampening of the raw data, especially when applied on a per gene basis.

In instances where data stemming from different probe sets of a particular gene (either expression or SNP array) provides contradictory evidence, a platform-specific summary category of ‘undetermined’ is given, signifying that evidence for both gains or losses are present for the particular gene. During the evaluation of the cross platform category, ‘undetermined’ will be used as a wildcard for drawing concordance/ discordance conclusions.

### Cross platform data agreement

After each platform has been evaluated for all platform-specific summary categories, an additional evaluation is performed to provide a cross platform (‘Overlay’) category. When agreement is reached between the platforms, an overall category signifying the overall trend is generated for the gene, and thus, used to highlight the data point in aCGHViewer ^©^. Due to the differences in the nature of the overlay, the highlighted colors (red or green) require different interpretation (Table 3).

In all other instances, where there is either (a) no agreement between the platforms, (b) data points which do not surpass the user-defined thresholds (for non-categorized data), or (c) the gene is only covered by one of the platforms, a category value of ‘0’ is assigned, signifying that no agreement can be reached. The resulting data points in these cases will be displayed as black dots on aCGHViewer^©^.

### Details of the evaluation methods

For genes with single data points, all of the evaluation methods will generate the same result. However, when a gene is represented by multiple probes (probe sets), the results could differ slightly depending on which evaluation method is selected. To show the subtle nuisances of the various evaluation methods, we have undertaken a preliminary analysis for expression data of one gene for user reference. As an example, analysis of the *EGFR* gene demonstrates the changes that might be encountered using the different calculation options.

**Figure 7.**
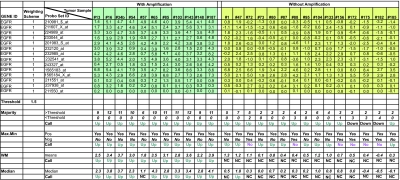
A preliminary evaluation of the differences in calculation methods and their subsequent determination of the platform specific category for the *EGFR* gene. The expression of *EGFR* is interrogated by multiple probe sets on the Affymetrix Gene-Chip U133 Plus 2 array. Samples were segregated by the presence or absence of *EGFR* amplification as seen on the BAC arrays (data not shown). Notice in tumor samples with *EGFR* amplification, all of the evaluation methods show upregulation of *EGFR* expression, regardless of the choice of calculation method (with the exception on the ‘median’ approach on tumor #57). Where there is no *EGFR* amplification, the choice of calculation method has a large impact as to whether the platform specific category is called up, down or no change. However, in the overlay of the aCGH platform with expression, those tumor samples without amplification would yield a cross platform category of “no agreement” and thus would not be highlighted in the overlay.

Thus, *EGFR* is represented by multiple probe sets on the Affymetrix U133Plus2 platform. Using SAM, a two-class unpaired analysis (using a t-test as test statistic with 5,000 permutations) was performed among samples with and without evidence of copy number amplification of the *EGFR* locus using the BAC array data (Rossi et al. 2005). All probe sets were weighted equally and a threshold value of 1.5 (SLR) was chosen for this analysis (see Fig. 7). All of the methods used for this analysis, regardless of stringency, gave the same result for samples that showed a physical amplification, with the exception of one tumor sample (#57). Analysis of tumors with no amplification, however, showed a distinct difference between the more liberal evaluation methods (‘majority’ and ‘max-min’) and the more stringent evaluation methods (‘weighted means’ and ‘median’). The results from the ‘majority’ and ‘max-min’ evaluation methods indicate that there is, in general, an increase in expression, even though there is no amplification. Using the ‘weighted means’ and ‘median’ evaluation methods, increase in expression in any of the samples without physical amplification is not detected. However, by weighting the different probe set based on their suffixes may introduce additional changes when generating the platform specific category (see Fig. 7).

The biological significance of the data-analysis variation can be explained in one of two ways: either there is an upregulation of gene transcript levels without physical amplification of the gene, or the categories made by the Overlay Tool^©^ represent false discoveries due to the stringency of the evaluation method. As such, it might be expected that the false discovery rate would decrease with a more stringent evaluation method.

### Data input requirements

The Overlay Tool^©^ accepts either a 4- or 5- column tab-delimited data set, with an optional fifth column to include categorical information from prior statistical analyses. Only values of 3 (signifying gain or upregulation), 1 (signifying no change) and 2 (signifying loss or downregulation) are acceptable. The first column contains the gene symbol; the second column contains the chromosome number information; the third column contains the mapping location of the gene; and the fourth column contains the value of the particular platform. If the delimited text file can be displayed with the aCGHViewer^©^, the formatting will be acceptable to the Overlay Tool^©^. Two sample data files (6K_BAC_array_test_data.txt and U133Plus2_test_data.txt) are included in the self-extracting zip file for formatting reference.

### Data output

Currently, the Overlay Tool^©^ outputs tab-delimited text files for visualization with the option to save the analysis as an .olt file. In addition to including gene symbol, the chromosome location and the chosen primary display value, the Overlay Tool^©^ adds an additional column of data using the cross platform category data generated as a result of ‘cross platform data agreement’ processing. This category data is then used to highlight changes that stem from agreement between the two platforms and utilizes the flagging capacity of the aCGHViewer^©^ to display the data points as red or green (Fig. 3b and 3c).

## Discussion

As high throughput, genome-wide screening using microarray technology becomes more common, the understanding of the underlying genetics of cancer is expected to increase exponentially. While aCGH and gene expression profiling have independently contributed to this understanding, additional information can be mined and extracted through the complementary use of these high throughput platforms. By overlaying the datasets between two or more platforms, gene candidates can be quickly identified and prioritized for further biological studies. To that end, we have devised an approach for performing the overlay of two or more datasets by combining the data using a gene-centric approach, as well as developing software to facilitate such an overlay in a time-efficient manner. Once the data sources for two or more platforms are identified, and all the necessary options for launching the Overlay Tool^©^ are selected, it will then perform an overlay of the datasets. The aCGHViewer^©^ can be used to visualize the results of the overlay in addition to a summary user-interface for more in-depth investigation of the Overlay Tool^©^ algorithm. The inherent flagging mechanism in aCGHViewer^©^ allows potential candidate genes to be easily identified due to the data point highlighting of the overlay whenever a concordance is reached between two datasets (i.e. amplification in aCGH with overexpression in gene expression). Although the development of this application was facilitated by specifically using the RPCI BAC arrays, Affymetrix GeneChip ^®^ expression arrays and Affymetrix GeneChip ^®^ mapping SNP arrays, additional custom platforms can easily be included in the Overlay Tool^©^ through the Custom Array Platform adding function included in the current release. Thus, whenever the annotation file can be formatted in a way that is consistent with the other annotation files provided with the Overlay Tool^©^, any platform that can be evaluated using the calculation options described above can be processed.

## Materials and Methods

Figure 5 shows the workflow of the RPCI Overlay Tool. The program has been implemented in Microsoft^®^ Visual Basic 2005^®^ using an object-oriented approach to facilitate future developments. The basic components of the RPCI Overlay Tool^©^ include the graphical user interface (GUI) and summary interface. After the overlay analysis is complete, the aCGHviewer can be invoked via the summary interface to connect to public databases such as NCBI or UCSC genome browser for additional information of gene targets. The results of the overlay analysis are outputted into a tab-delimited text file for user reference.

## Availability

RPCI Overlay Tool^©^ is freely available for academic users for non-commercial purposes at http://falcon.roswellpark.org.

## Figures and Tables

**Table 1.** Summary of different array identifiers for the EGFR gene.

**Experiment Type**	**aCGH**	**Expression Profile**	**SNP Studies**
*Array Platform*	*RPCI 6k BAC Array*	*U133 Plus 2 Array*	*Mapping 100K Array*
EGFR	RP11-81B20	1565 483_at	SNP_A-1656548
	RP11-89E8	1565 484_X_at	SNP_A-1656756
		201983_s_at	SNP_A-1663463
		201984_s_at	SNP_A-1665473
		211550_at	SNP_A-1703938
		211551_at≠	SNP_A-1705242≠

≠Since there were 15 probe sets for EGFR on the Affymetrix U133 Plus 2 array, many of these have been omitted to abbreviate the table.

≠Since there were 29 SNPs for EGFR on the Affymetrix Mapping 100K array, the ‘majority’ of have been omitted to abbreviate the table.

**Table 2.** Values obtained from two high throughput platforms for the EGFR gene.

**Experiment Type**	**aCGH Studies**		**Expression Studies**	
*Array Platform*	*RPCI 6K BAC Array*	Log*_2_Ratio*	*U133 Plus 2 Array*	*SLR*
EGFR	RP11-81B20	4.41	1565483_at	4.10
	RP11-89E8	4.425	1565484_x_at	2.93
			201983_s_at	4.49
			201984_s_at	4.67
			211550_at	−0.04
			211551_at	0.40
			211607_x_at	4.74
			224999_at	4.66
			232120_at	3.24
			232541_at	4.42
			232925_at	4.58
			233044_at	2.90
			237938_at	1.62
			243327_at	0.45

**Table 3.** A brief description of the highlighting convention used for displaying correlations in aCCHViewer using output from the overlay tool. Based on the choice of the overlay type, the colors used to highlight concordance may signify different types of agreement. This is especially evident for the overlay of aCGH with LOH.

**Overlay Type**	**Data Points highlights in aCGHViewer**		
	**Red**	**Green**	**Black**
aCGH/Expression	Gain/Upregulation	Loss/Downregulation	No agreement. not coverd by all platforms, no change
SNP CN/Expession	Gain/Upregulation	Loss/Downregulation	No agreement. not coverd by all platforms, no change
aCGH/SNP LOH/Expression	Gain/Upregulation/LOH	Loss/Downregulation/LOH	No agreement. not coverd by all platforms, no change
SNP LOH/Expression	LOH/Upregulation	LOH/Downregulation	No agreement. not coverd by all platforms, no change
aCGH/SNP LOH	Loss or Gain/LOH	No Loss or no Gain/LOH	No LOH

## References

[b1-cin-03-307] Carvalho B, Ouwerkerk E, Meijer GA (2004). High resolution microarray comparative genomic hybridisation analysis using spotted oligonucleotides. J. Clin. Pathol.

[b2-cin-03-307] Cui X, Churchill GA (2003). Statistical tests for differential expression in cDNA microarray experiments. Genome Biol.

[b3-cin-03-307] Daruwala RS, Rudra A, Ostrer H (2004). A versatile statistical analysis algorithm to detect genome copy number variation. Proc Natl. Acad. Sci., U.S.A.

[b4-cin-03-307] Eilers PH, de Menezes RX (2005). Quantile smoothing of array CGH data. Bioinformatics.

[b5-cin-03-307] Fridlyand J, Snijders AM, Pinkel D (2004). Hidden Markov models approach to the analysis of array CGH data. Journal of Multivariate Analysis.

[b6-cin-03-307] Hupe P, Stransky N, Thiery JP (2004). Analysis of array CGH data: from signal ratio to gain and loss of DNA regions. Bioinformatics.

[b7-cin-03-307] Iafrate AJ, Feuk L, Rivera MN (2004). Detection of large-scale variation in the human genome. Nat. Genet.

[b8-cin-03-307] Jong K, Marchiori E, Meijer G (2004). Breakpoint identification and smoothing of array comparative genomic hybridization data. Bioinformatics.

[b9-cin-03-307] Kallioniemi A, Kallioniemi OP, Sudar D (1992). Comparative genomic hybridization for molecular cytogenetic analysis of solid tumors. Science.

[b10-cin-03-307] Olshen AB, Venkatraman ES, Lucito R (2004). Circular binary segmentation for the analysis of array-based DNA copy number data. Biostatistics.

[b11-cin-03-307] Rossi MR, La Duca J, Matsui S (2005). Novel amplicons on the short arm of chromosome 7 identified using high resolution array CGH contain overexpressed genes in addition to *EGFR* in glioblastoma multiforme. Genes Chromosomes Cancer.

[b12-cin-03-307] Sandberg AA (1980). Chromosomes and causation of human cancer and leukemia: XL. The Ph1 and other translocations in CML. Cancer.

[b13-cin-03-307] Shankar G, Rossi MR, MacQuaid DE (2006). aCGHViewer©: A Generic Visualization Tool For aCGH data. Cancer Informatics.

[b14-cin-03-307] Tusher VG, Tibshirani R, Chu G (2001). Significance analysis of microarrays applied to the ionizing radiation response. Proc. Natl. Acad. Sci. U.S.A.

[b15-cin-03-307] Wang P, Kim Y, Pollack J (2005). A method for calling gains and losses in array CGH data. Biostatistics.

[b16-cin-03-307] Yeung KY, Ruzzo WL (2001). Principal component analysis for clustering gene expression data. Bioinformatics.

